# Foreign body‐associated endobronchial inflammatory polyps

**DOI:** 10.1002/ccr3.1605

**Published:** 2018-06-05

**Authors:** Masayoshi Higashiguchi, Ryutaro Jikuya, Hiromi Kimura, Tomoshige Matsumoto, Takashi Fujii

**Affiliations:** ^1^ Department of Internal Medicine Osaka Anti‐Tuberculosis Association Osaka Hospital Neyagawa City, Osaka Japan

**Keywords:** endobronchial inflammatory polyp, foreign body

## Abstract

Endobronchial polypoid lesions can be observed after removal of a foreign body and usually regress without treatment. Bronchial obstruction with a foreign body can cause atelectasis in nonelderly adults without history of an episode of aspiration.

## CASE DESCRIPTION

**Figure 1:**
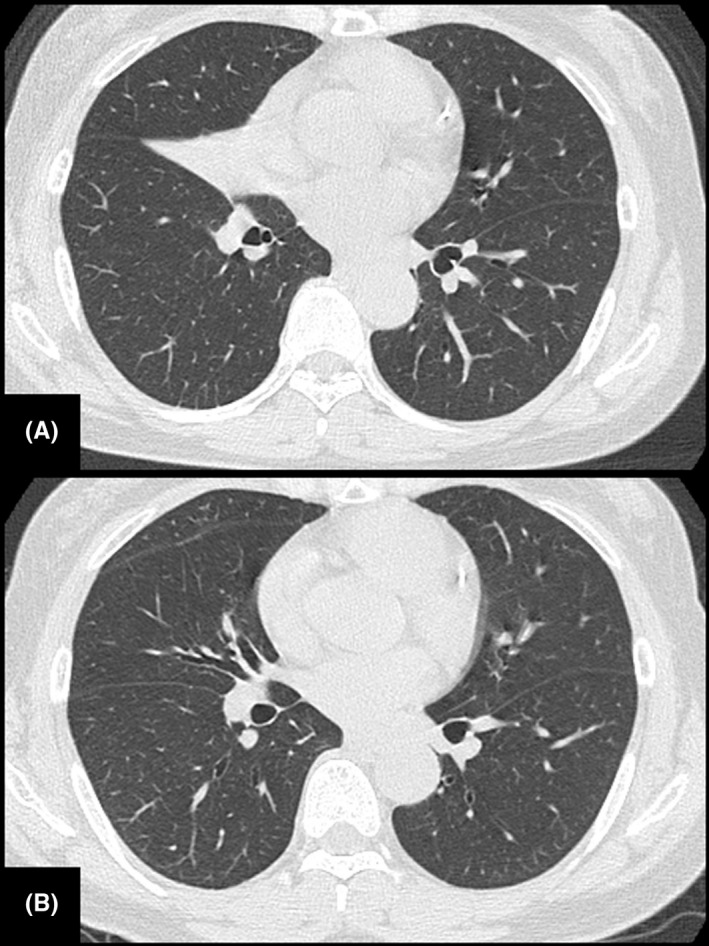
(A) Chest CT showed atelectasis of the middle lobe of the right lung. (B) The atelectasis resolved after the removal of a foreign body in the right middle lobe bronchus.

**Figure 2:**
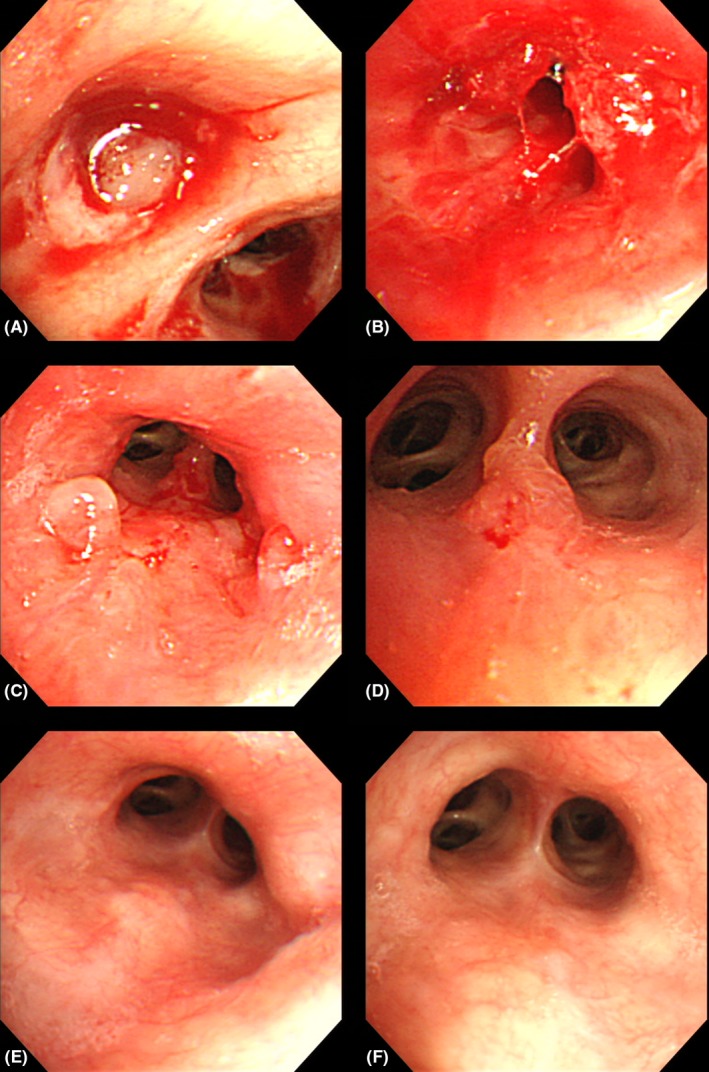
(A, B) Bronchoscopy revealed that the right middle lobe bronchus was occluded by a foreign body which was subsequently removed bronchoscopically. (C, D) Several polypoid lesions were observed in the middle lobe bronchus after the removal of the foreign body. (E, F) The polypoid lesions regressed spontaneously.

**Figure 3:**
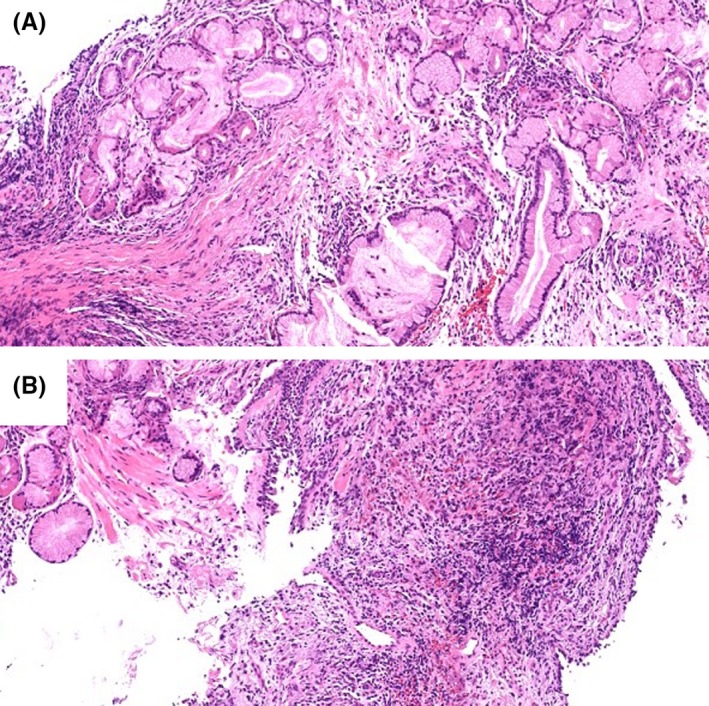
(A, B) Histopathological examination of the polypoid lesions (hematoxylin and eosin stain).

A 59‐year‐old woman was referred to our hospital because an abnormal shadow was found in the right middle lung field in a chest X‐ray taken during a routine medical checkup. Her medical history was unremarkable, including no history of cerebrovascular disease or neuromuscular disease. Chest CT showed atelectasis of the middle lobe of the right lung (Figure 1A). Bronchoscopy revealed that the right middle lobe bronchus was occluded by a foreign body (Figure 2A). The foreign body was removed bronchoscopically, but the bronchial mucosa could not be examined adequately during the initial procedure due to substantial bleeding (Figure 2B). The foreign body turned out to be of plant origin and was considered to be a piece of vegetable although the patient did not remember having experienced an episode of aspiration. Seven days later, chest CT showed that the atelectasis had resolved (Figure 1B). On the same day, bronchoscopy revealed several polypoid lesions in the middle lobe bronchus (Figure 2C,D). The histopathological examination of the biopsy specimens revealed hyperplasia of the bronchial glands and nonspecific granulomatous inflammation, findings which were compatible with the diagnosis of inflammatory polyps (Figure 3A,B). Eight weeks later, the polypoid lesions were observed to have regressed spontaneously (Figure 2E,F).

Endobronchial polypoid lesions can be observed after removal of a foreign body and usually regress without treatment.^1^ Some previous reports suggested that corticosteroid therapy may facilitate regression of endobronchial inflammatory polyps.^2^, ^3^, ^4^

## CONFLICT OF INTEREST

None declared.

## AUTHORSHIP

MH: was the primary doctor of the patient. MH, RJ, and HK: performed bronchoscopy. MH: wrote the initial draft of the manuscript. All authors critically reviewed the manuscript and approved the final version of the manuscript.

## INFORMED CONSENT

Informed consent was obtained from the patient for publication of this case report.
